# Systematic Characterization and Analysis of the Freeze–Thaw Tolerance Gene Set in the Budding Yeast, *Saccharomyces cerevisiae*

**DOI:** 10.3390/ijms26052149

**Published:** 2025-02-27

**Authors:** Donovan Hartnett, Michael Dotto, Ashley Aguirre, Sophia Brandao, Melanie Chauca, Sandra Chiang, Madison Cronin, Niti Deokar, Autum Martin, Zuri McCune, Joseph Onwusogh, Arisbeth Paulino-Cruz, Angelina D. Gonzalez Soto, Aisha Swaray, Maxwell Verdiner, Majda Rayah, James T. Arnone

**Affiliations:** 1 Department of Biological and Environmental Sciences, Le Moyne College, Syracuse, NY 13214, USA; 2 W.E.B. du Bois Scholars Institute, Inc., Princeton, NJ 08542, USA

**Keywords:** freeze–thaw tolerance, gene expression, thermal tolerance, *Saccharomyces cerevisiae*

## Abstract

The budding yeast *Saccharomyces cerevisiae* is a widely utilized model system with myriad applications in terms of industrial, biotechnology, and synthetic biology purposes. One such application is the biosynthesis of commercially and medically important bioactive compounds and their precursors, which oftentimes require culturing conditions at low temperatures to optimize production yield rather than cellular fitness. To lend insight into genetic modifications that may assist this goal, this work focuses on a systematic analysis of the genes that result in an increase in survival following freezing. At present, these genes have been identified in a wide variety of *S. cerevisiae* wild-type backgrounds—that vary significantly in their properties and behaviors—and in the conditions that led to the annotation of the freeze–thaw survival phenotype. In this work, we report a complete characterization of the thermal tolerance and viability for the freeze–thaw gene family following a standardized protocol within a unified genetic background, the extensively used BY4741 laboratory strain. Our results reveal that five out of these six genes are linked to increased viability in response to both freeze–thaw stress as well as enhanced survival during a heat shock stressor. Follow-up analysis characterized the local spatial effects that gene modification at each locus causes when utilizing the common kanamycin resistance cassette (KanMX6) for the creation of mutant strains and engineering purposes.

## 1. Introduction

The budding yeast, *Saccharomyces cerevisiae*, is a single-cell model eukaryote that has been widely studied and is extensively utilized for its ability to produce commercial products that include breads and beers as well as an increasing number of valuable secondary metabolites with industrial applications, including biofuels and pharmaceuticals [1,2,3,4]. As a eukaryotic microbe, this organism offers many advantages, including a high-quality reference genome that has been studied for ~30 years, during which time it has been extensively annotated and refined [5,6]. This has led to a wealth of tools for genetic manipulation to produce both gene deletions and modifications via site-directed mutagenesis—including CRISPR-Cas9 and multiple selectable and counter-selectable markers—which resulted in extensive functional characterization of a genome that is compiled, continually updated, and readily accessible for all researchers at the *Saccharomyces* Genome Database [7,8,9,10,11].

The wealth of tools and resources available facilitates the genetic modification and engineering of *S. cerevisiae* for both functional studies as well as industrial applications through diverse and increasingly creative genetic and genomic manipulations [12]. This organism undergoes efficient homologous recombination (HR), offering the ability to produce stable manipulations directly to the chromosome. This ability has allowed researchers to employ HR for targeted mutagenesis that allows for the modulation of promoter strength that drives the level of transcription for an mRNA, that regulates the localization and secretion of a protein by the addition or modification of protein tags (e.g., a nuclear localization signal or a signal sequence, to name just two examples) [13,14]. There is also an increasing number of well-defined and cataloged resources that allow for the de novo construction of designer gene expression systems, with easily selected promoters (constitutive or inducible), terminator sequences, protein tags (for purification), and translational assemblies as complete units [15].

These features have been used in increasingly complex ways to bioengineer *Saccharomyces cerevisiae* for a variety of uses and applications. While the canonical application of this yeast for beer making and fermentation is still going strong, it is possible to genetically enhance this organism for improved characteristics—targeting changes that modulate ethanol, aromas, and flavors [16]. The applications extend far beyond products for human consumption to include complete biosynthetic pathways to produce medicines—such as the production of chemotherapeutic drugs, including Taxol and artemisinin; industrial products, including perfumes (α-santalene); and biofuel products including ethanol, butanol, alkanes, and fatty acids [17,18,19,20]. There are numerous examples that illustrate the potential to harness this kitchen staple, baker’s yeast and transform it into a versatile cell factory [21]. The fermentation process depends on the ability of *S. cerevisiae* to grow and survive when subjected to different conditions that are stressful to the organism but improve the ethanol production and yield [22]. One challenge faced (among many) is that the compounds that are introduced into this cell may have a negative impact on the cell’s fitness, and non-native pathways can be quite sensitive to stress factors, both of which can limit the desired output [23].

Additionally, chromosomal modification and integration—while much more stable than plasmids or extrachromosomal introduced pathways—can result in unintended consequences only visible upon non-optimal growth conditions [24]. There is an increasing body of evidence that there are functional relationships between clustered genes for the proper regulation of the production of primary and secondary metabolites [25,26]. These relationships have a functional component, where the expression of gene neighbors can depend on the activity of shared *cis* and *trans* regulatory factors and their proximity [27,28]. As a result, the target site of modification can result in significant differences in the transcriptional output, a finding that was initially identified at heterochromatin adjacent areas at the telomeres [29]. There are pervasive differences in expression output in identical genetic constructs that are evident across the *S. cerevisiae* genome [30,31]. The choice of integration construct can also exert a reciprocal effect and influence the expression of the genes flanking the integration target site. This can be significant enough for the modified yeast strain to behave phenotypically, as if the flanking gene, which was not modified in any way, was a null mutant [32,33].

There are numerous, well-characterized cellular changes that *S. cerevisiae* employs to manage the stress that is triggered by thermal fluctuations that allow it to maintain homeostasis. The process of freezing results in the formation of damaging ice crystals and denatured proteins, and this damage can be limited by the production of cellular osmolytes (e.g., trehalose) and chaperone proteins to improve proteostasis, respectively [34,35]. Additionally, alterations in protein half-life via changes in proteasome activity can also increase cellular fitness and survival under these stressors [36].

In this present work, we identify the gene set whose deletion from the genome yields a phenotype of increased viability and survival following a freeze–thaw stress compared to a wild-type strain. This led to a final list that included six genes: *AQY1*, *ATH1*, *CAR1*, *POG1*, *PUT1*, and *YCP4*. The annotated functions of these genes varied, two of these code for proteins that are involved in maintaining homeostasis during osmotic shock by regulating water transport and trehalase (*AQY1* and *ATH1*), an arginase (*CAR1*), a transcription factor (*POG1*), and two mitochondrial linked genes (*PUT1* and *YCP4*). Interestingly, these genes have been identified in several different genetic backgrounds of *Saccharomyces cerevisiae*—which can have surprisingly different behaviors—under freezing conditions and durations that varied considerably.

Here, we performed a phenotypic screen of this gene set to investigate its thermal sensitivity in the BY4741 strain of *S. cerevisiae* (a widely used haploid strain) using a standardized protocol. We mapped each member of this freeze–thaw gene set at each locus, and performed a computational analysis to characterize the gene expression patterns across each region throughout the cell cycle and during the induction of the environmental stress response through multiple mechanisms. The effects following the integration of the widely used (and highly expressed) kanamycin resistance (KanMX6) gene were tested to characterize potential secondary effects for targeted integration at each site, and gene expression analysis was performed at the most sensitive locus. Here, we provide a comprehensive, systematic analysis of the freeze–thaw gene set that can be used as a guide for researchers looking for genetic manipulations to alter cellular viability and fitness under temperature deviations, as well as the evaluation of the effect of the integration of highly transcribed genetic constructs into each of these genetic loci.

## 2. Results and Discussion

### 2.1. Identification of Non-Essential Genes That Increase Viability Following Freezing

The set of genes that results in increased survival and freeze–thaw tolerance upon their deletion was accessed and downloaded from the *Saccharomyces* genome database [37]. This resulted in the identification of six genes with a variety of annotated molecular and cellular functions, which have been characterized in a variety of different *Saccharomyces* genetic backgrounds (Table 1). A review of the conditions used in the characterization of each gene that has been described throughout the literature revealed significant differences in the protocols used, specifically the duration and the exact details of the freezing and thawing process. Our experimental design was based on comparing the freeze–thaw survival and viability at two time points—48 h of freezing and 144 h of freezing—which represented a range that ‘bookends’ the published studies, both the shortest and the longest screens that have been analyzed (full details of our protocol can be found in the Materials and Methods section) [38,39].

### 2.2. Characterization of Tolerance to Freezing Within a Uniform Genetic Background Following a Standardized Protocol

To test what effect the deletion of each identified gene would have within the widely used *Saccharomyces cerevisiae* laboratory strain BY4741, survival was tested following freezing for a short (two-day, 48 h) and an intermediate (six-day, 144 h) incubation at either −20 °C or −80 °C without the presence of any cryoprotectants (e.g., glycerol, DMSO, sucrose, etc.) [46]. In addition, each strain was also screened for survival when incubated at an elevated temperature (40 °C), with growth and viability compared to the wild-type genetic background which exhibits reduced viability. This approach would allow for the complete characterization of thermal tolerance—to both elevated and reduced temperatures—following a standard set of conditions to allow for direct comparison between strains.

**Figure 1:**
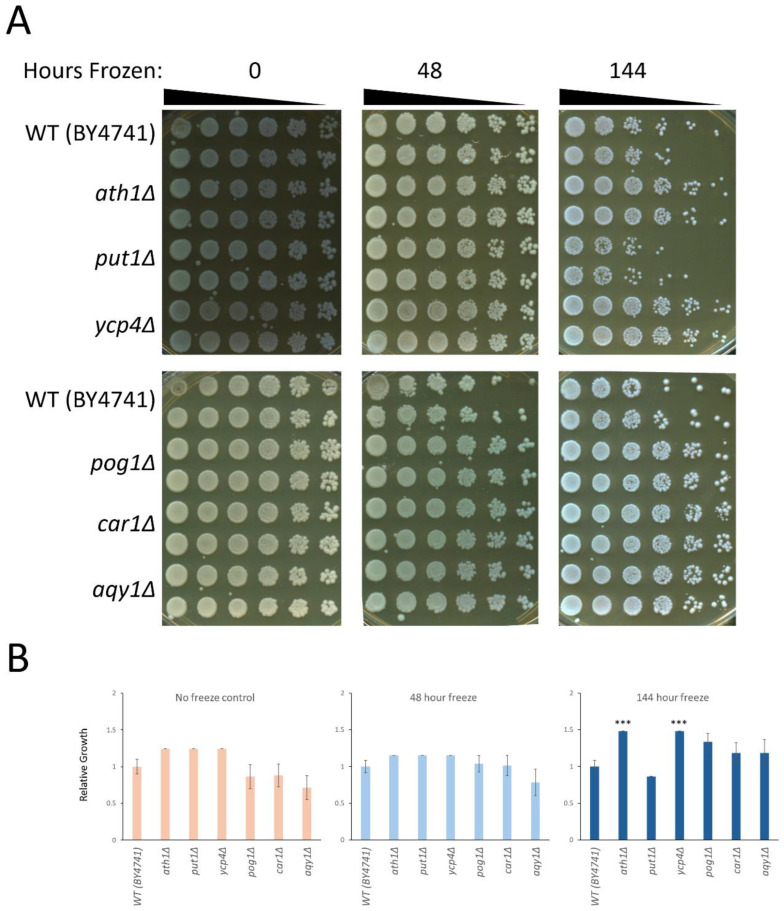
The growth analysis of the freeze–thaw phenotype seen at −20 °C in the BY4741 genetic background. Saturated cultures of yeast were washed and resuspended in ddH_2_O, and frozen for the indicated periods of time before being thawed, serially diluted, and plated on enriched media. (**A**) Representative plates depicting the growth of each strain performed in duplicate (each strain is plated as two rows, representing technical replicates). (**B**) The quantification of the growth phenotypes seen for multiple biological (*n* = 3) and technical (*n* = 6) replicates were averaged and plotted in the graphs. Error bars represent the S.E.M. Significant differences were determined by a Student’s *t*-test, comparing the growth of each mutant to the growth of the WT strain and are indicated on the graph (*** = *p* < 0.05).

Saturated cultures of yeast were washed and resuspended in ddH_2_O and were then frozen for the indicated time periods prior to thawing and performing growth analysis. The growth observed at zero hours of freezing (the no-freezing control condition) was consistent, and comparable levels of growth were observed for the wild type and for each of the mutants tested (Figure 1A, left panels). Freezing the cells to −20 °C and incubation for 48 h prior to thawing did not result in an observable change in the growth of any of the mutant strains relative to the wild-type strain (Figure 1A, middle panels). Extending the time that the cultures were frozen to 144 h at −20 °C prior to thawing and analysis resulted in a noticeable decrease in the survival of the wild-type strain, and the appearance of resistance within several of the mutant strains tested (Figure 1A, right panels). Quantification of the growth phenotypes across triplicate experiments confirmed these observations—there was no statistically significant difference that was seen in the control or 48 h frozen cultures; however, there was an increase in the growth of several mutants relative to the wild-type strain seen for 144 h of freezing (Figure 1B). Both *ATH1* and *YCP4* exhibited a statistically significant increase in growth compared to the growth of the wild-type strain. In addition, three strains showed a subtle increase in growth that was reproducible, but did not cross the threshold of statistical significance: *POG1*, *CAR1*, and *AQY1*. This was consistent with a previously published characterization of these strains, as the relative changes were quite subtle for several of the identified genes. Only the *PUT1* mutant strain demonstrated no change in growth following freezing under these conditions compared to the wild-type strain. This particular result most likely is the result of strain-specific differences, the sensitivity and resolution of the growth assay employed in our study, or—most likely—a combination of both factors.

The sensitivity of each strain was then determined by freezing to a temperature of −80 °C for incubation and the determination of survivability. There were no observable differences in growth seen following 0 h or 48 h of freezing (Supplemental Figure S1A, left and middle panels)—in agreement with the results obtained for a freezing temperature of −20 C. Surprisingly, there was no noticeable difference seen following 144 h of incubation at −20 °C (Supplemental Figure S1A, right panel). Analysis of multiple replicates verified these observations; there was a reproducible, albeit nominal, increase in survivability seen at 144 h of freezing at −80 °C in several of the genetic backgrounds. None of these observations crossed the threshold to a statistically significant difference in growth, however. This interpretation lends support to and validates the link to enhanced viability at freezing temperatures for five of the six genes tested (all except for *PUT1*), extending this phenotype into this genetic background. The differences seen between the −20 °C and −80 °C temperatures are likely the speed at which the strains were frozen, limiting the potential cellular damage that occurs from the formation of ice crystals.

### 2.3. Complete Characterization of Thermal Tolerance Within the Freeze–Thaw Gene Family

**Figure 2:**
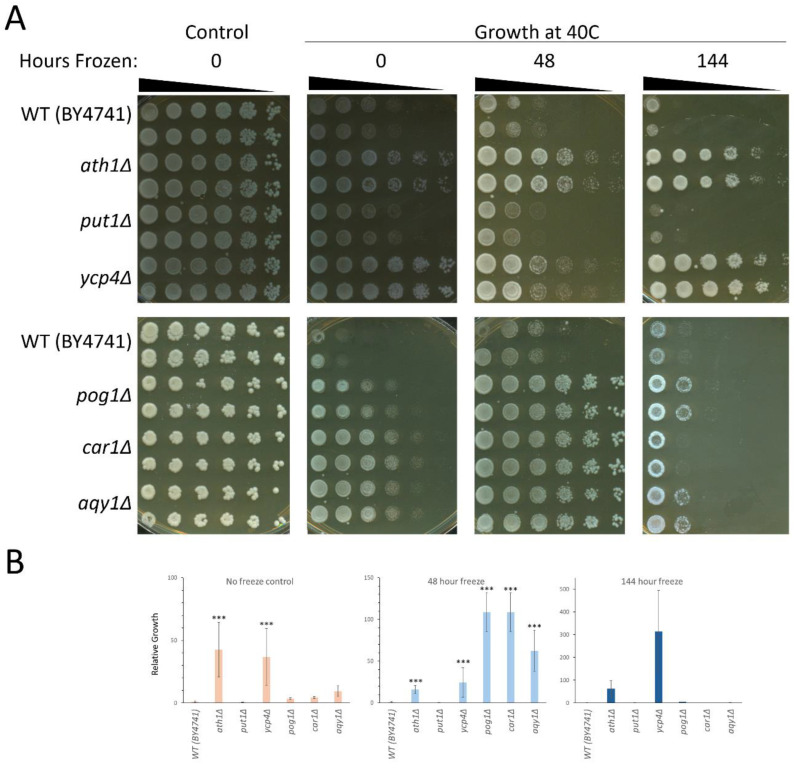
Comprehensive growth analysis of thermal sensitivity phenotypes in the BY4741 genetic background. Strains were incubated and growth was assayed at either optimal (control, 30 °C) or elevated (40 °C) temperatures with and without freezing and incubation at −20 °C. (**A**) Representative plates depicting the growth of each strain performed in duplicate (each strain is plated as two rows representing technical replicates). (**B**) The quantification of the growth phenotypes seen for multiple biological (*n* = 3) and technical (*n* = 6) replicates were averaged and plotted in the graphs. Error bars represent the S.E.M. Significant differences were determined by a Student’s T-test, comparing the growth of each mutant to the growth of the WT strain. Significant differences in the growth observed are indicated on the graph (*** = *p* < 0.05).

Upon the completion of testing the phenotypes for each member of the gene family that is linked to freeze–thaw tolerance, our focus shifted to a broader characterization of thermal tolerance. To accomplish this, cultures were grown at an elevated temperature to induce the heat shock stress response in *Saccharomyces cerevisiae* [47]. This was accomplished via incubation at 40 °C and was performed in both the presence and the absence of an incubation at freezing temperatures as performed above. When grown at an optimal temperature, 30 °C, each of the mutant strains grew at a rate that was indistinguishable from the growth that was observed in the wild-type strain (Figure 2A, left panels). Incubation at a temperature of 40 °C, without any freezing incubation beforehand, showed a sharp decrease in viability seen in all strains; however, two mutants demonstrated increased growth relative to the wild-type strain, both the *ATH1* and *YCP4* null strains (Figure 2A, second panel from the left).

When each strain was incubated at −20 °C for either 48 h or 144 h prior to growth at 40 °C, several mutant strains demonstrated increased survival compared to the wild-type (Figure 2A, the right two panels). Upon quantification, we observed that the deletion of either *ATH1* or *YCP4* results in a strong, statistically significant resistance to heat sensitivity (Figure 2B, left graph). Incubation at a temperature of −20 °C for 48 h prior to subjecting each strain to growth at 40 °C revealed that the deletion of *ATH1*, *POG1*, *CAR1*, *AQY1*, and *YCP4* increases survival significantly (Figure 2B, middle panel). Survival at 40 °C following incubation at −20 °C for 144 h varied widely; however, there was no consistent, reproducible change in viability seen in any of the mutant strains relative to the wild-type control (Figure 2B, right panel).

The same experimental design was tested with the freezing incubation at −80 °C. There was no difference seen in the growth of these strains at 30 °C, and as previously noted the deletion of either *ATH1* or *YCP4* confers increased viability when exposed to a consistent 40 °C heat shock for growth (Supplemental Figure S2). Upon 48 h of incubation at −80 °C, followed by growth at 40 °C, there was a marked decrease in viability; however, *ATH1*, *CAR1*, and *AQY1* each exhibited a significant increase in survival compared to the wild-type strain (Supplemental Figure S2B, center panel). Extending the time spent at −80 °C to 144 h prior to incubation at 40 °C decreased the survival of all strains—under these conditions, there were no observed differences that could be attributed to any of the genetic backgrounds tested (Supplemental Figure S2B, right panel).

### 2.4. A Map of the Genomic Distribution of the Freeze–Thaw Gene Family and Each Locus

**Figure 3:**
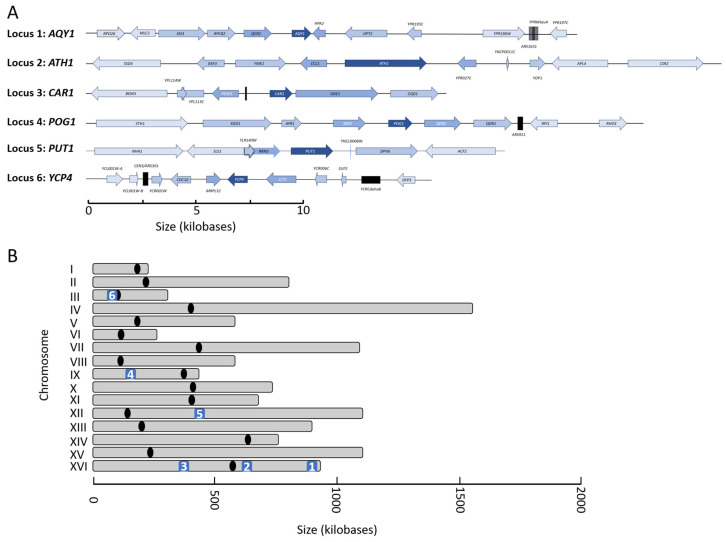
The genomic loci and chromosomal distribution of the freeze–thaw gene family in *Saccharomyces cerevisiae*. (**A**) The genomic neighborhood for each of the six genes that comprise the freeze–thaw gene family was investigated, and a map was generated for each locus. The freeze–thaw gene is annotated in the darkest blue color, and the neighboring genes that flank each target are colored with increasingly lighter shades of blue as the distance increases from the target gene. Non-open reading frame genomic features are identified with a black box where appropriate. (**B**) Each of the six loci is marked by an Arabic numeral that corresponds to the designation from part (**A**). The centromere for each chromosome is marked with a black oval.

Following the characterization of the thermal tolerance and phenotypes that are associated with this gene family, we mapped the genomic region surrounding all six loci and noted their genomic distribution (Figure 3). There have been a number of studies that have identified that a significant number of functionally related genes can be found as clusters interspersed throughout the genome in *Saccharomyces cerevisiae* [25]. Upon mapping each gene to its target neighborhood, there was no incidence of clustering seen within this gene set (Figure 3A). On a chromosomal level, there was no readily identifiable pattern attributed to this gene family (Figure 3B). Three genes within the freeze–thaw set all map to Chromosome XVI; however, their spacing throughout the chromosome is distant enough to prevent an interconnected transcriptional relationship. *YCP4* is located proximally to a centromeric repeat region in Chromosome III. *AQY1* is positioned closest to a telomeric region; however, it is separated from any repeats by several open reading frames. The other four genes, *ATH1*, *CAR1*, *POG1*, and *PUT1,* are in gene-rich areas that do not contain any notable features.

### 2.5. Transcriptional Analysis and Correlation Throughout the Cell Cycle and Stress Responses

**Figure 4:**
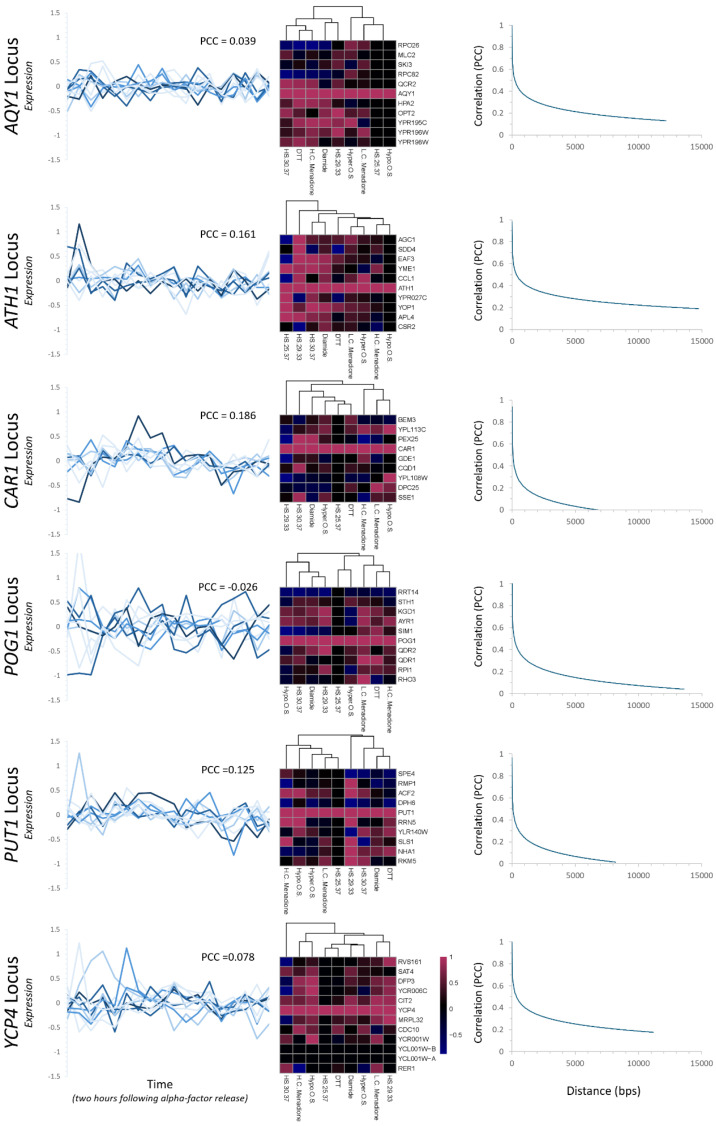
Transcriptional analysis across each genomic neighborhood, throughout the cell cycle and upon induction of the stress response. *Left panels:* Microarray expression profiles were accessed and the transcription of each member of the freeze–thaw gene set—and their neighbors that flank them on each side—were plotted for cycling cells. The color scheme is the same as seen in the locus maps in Figure 3. The Pearson correlation coefficient (PCC) was calculated for the entire locus and is indicated on each graph. *Middle panels:* The microarray expression profiles were accessed following the induction of nine different stressors. The PCC was calculated for each freeze–thaw gene compared to each one of the flanking neighboring genes, and the values are depicted as heatmaps. The target for comparison is located at the center (and has a PCC = 1 due to self-comparison). Flanking genes are kept in order (the *y*-axis), but the stressors are not (the *x*-axis) as hierarchical clustering was used to facilitate the identification of patterns. *Right panels:* The PCC was plotted as a function of distance comparing each freeze–thaw gene to the neighbors flanking it on either side. Graphs depict the plot of the decay curve relative to all PCC comparisons to the target gene at each locus. Heatmaps were generated in R, graphs were generated in Excel. (Abbreviations: HS29.33 = heat shock following a temperature shift from 29 to 33 °C, HS30.37 = heat shock following a temperature shift from 30 to 37 °C, HS25.37 = heat shock following a temperature shift from 25 to 37 °C, H.C. Menadione = high concentration of menadione, L.C. Menadione = low concentration of menadione, Hyper O.S. = hyperosmotic shock, and Hypo O.S. = hypoosmotic shock).

Transcription was characterized by extracting expression profiles for each of the freeze–thaw genes and their surrounding neighbors for computational analysis. It has been previously reported that there is a global, positive correlation between all genes and their proximal neighbors that decays in a manner that scales to the genomic distance between any two points [48]. This finding was identified within the budding yeast, *S. cerevisiae*, although it was also identified as conserved in broadly divergent eukaryotes—up to and including humans. Notably, there was a significant amount of variance in the degree of correlation and the rate of the decay of this correlation based on genomic location. To characterize this effect and measure it at each of the six loci for the genes within our study, we utilized this approach. This initial analysis focused on microarray datasets for cycling cells (cell cultures were synchronized by alpha-factor arrest, which blocks cell cycle progression at the G1/S transition). Measurements are plotted for each locus graphically, with the zero-minute mark indicating release from arrest (Figure 4, left panel). From this expression data, the average Pearson’s correlation coefficient was calculated throughout the cell cycle from this synchronized population. Five out of the six loci exhibited a weakly positive correlation throughout the measured time course—these were *AQY1*, *ATH1*, *CAR1*, *PUT1*, and *YCP4*. The *POG1* locus was the only outlier, which exhibited a weakly negative PCC anticorrelation under these conditions. The locus with the strongest correlation was the *CAR1* genomic region (PCC = 0.186), which was followed closely by the *ATH1* genomic region (PCC = 0.161). Altogether there was a positive—albeit weak—correlation at five of the six loci under steady-state cycling conditions.

As the process of freezing followed by thawing without the presence of a cryoprotectant is a type of stress, we expanded our computational analysis tracking the transcriptional changes at each locus by utilizing the environmental stress response microarray datasets [47]. Gene expression profiles for each member of the freeze–thaw gene family—and the neighboring genes that flank them on either side of the chromosome—were extracted and the correlation was calculated between each freeze–thaw gene and each neighboring gene. This analysis was completed over a variety of different conditions that induce broad transcriptional changes necessary for cellular survival and adaptation to changing environmental conditions. The correlation of transcription was visualized by heatmap to facilitate the identification of patterns (Figure 4, middle panels).

This analysis revealed that within each genomic neighborhood the correlation between transcription varied based on exposure to specific stressor conditions at each locus. Analysis of the heatmaps that were generated reveals that overall, there appears to be a non-random, positive correlation that can be seen across each region in response to specific stressors. Some of the relationships that demonstrated the strongest, positive correlation in transcription include: *AQY1* and its neighbor *QCR2* (30–37 °C heat shock, high-concentration menadione, and DTT exposure), *ATH1* and its neighbor *CCL1* (hyperosmotic shock, 29–33 °C heat shock), *CAR1* and its neighbor *PEX25* (30–37 °C heat shock, diamide exposure), *PUT1* and its neighbor *RRN5* (29–33 °C heat shock and hypoosmotic shock), and *YCP4* and its neighbor *CIT2* (29–33 °C heat shock, low-concentration menadione, and hypoosmotic shock). Throughout each of the analyzed loci, the transcriptional responses varied from a combination of tightly correlated positive transcriptional relationships (PCC > 0.5) to significant anticorrelation relationships and to non-correlation at all (PCC = 0). Combining our results with previously published work can be reconciled as follows: potential interconnected relationships across any given locus that exert spatial and positional effects that could affect transcription can only be seen under specific growth conditions.

To obtain a more complete picture of the inter-related interactions that might be present at each region, the calculated PCCs for each gene were plotted as a function of genomic distance relative to the freeze–thaw gene and a decay curve was fit to the datasets (Figure 4, right panels). Previous global analysis has shown that there is a non-random, positive correlation that is observed in *Saccharomyces cerevisiae* that varies significantly in strength and the distance of correlation seen globally [48]. At each locus, there is a positive correlation that varies in terms of the strength and distance of the decay function. The three loci with the strongest correlation and effect are *AQY1*, *ATH1,* and *YCP4*, while the weakest correlations with the quickest decay are *CAR1*, *POG1*, and *PUT1*.

### 2.6. Screening for Local Interactions and Spatial Position Effects

As there are many potential biotechnological applications for *Saccharomyces cerevisiae* that could potentially benefit from the enhanced viability and survival at the thermal ranges that have been described previously, and the observation that there is a positive correlation in transcription seen at each of these loci, we set about to determine whether the genomic modification within these loci may be subject to ‘Neighboring Gene Effects’ [24,32,33].

The budding yeast ‘knock-out’ collections (YKOCs) are a resource representing several collections that together contain over 21,000 yeast deletion mutant strains that have been used extensively by researchers and have led to numerous valuable studies and insights since their creation [49,50]. The construction of these collections came from the precise integration of the KanMX6 coding sequence to replace the coding sequence of a specific coding sequence and conferring antibiotic resistance to facilitate the selection of the mutants. This specific modification results in an extremely highly expressed transcript at the integration site—which can result in localized disruption to the expression of neighboring genes that leads to observable phenotypes.

Each of the genes that flank each member of the freeze–thaw gene set was performed and is summarized in Table 2. A review of the annotated phenotypes revealed that three of the genes within this set have a documented growth change during the response to an increased temperature. *RRN5* (a transcription factor for RNA pol I) is an essential gene, so it cannot be deleted to test its null phenotype; however, a conditional mutant strain has been shown to exhibit increased sensitivity to thermal stress than a wild-type strain [51,52]. *MRPL32* (a structural component of the mitochondrial ribosome) also exhibits increased sensitivity in a homozygous diploid null mutant strain [53]. The *CIT2* (*Citrate synthase*) gene exhibits a decreased sensitivity to increased heat, which was identified by a brief exposure to a very high (62 °C) thermal temperature [54].

The other nine genes that flank these genes have no annotated thermal sensitivity (the annotated function is provided in the parenthetical following each gene): *HPA1* (*a subunit of Elongator complex*), *QCR2* (a mitochondrial complex III component), *YPR027C* (unknown function), *CCL1* (a cyclin partner of the CDK Kin28p), PEX25 (a peroxisomal membrane peroxin), *GDE1* (Glycerophosphocholine (GroPCho) phosphodiesterase), *SIM1* (an SUN family protein), *QDR2* (a plasma membrane transporter), and *DPH6* (Diphthamide synthetase). As any modification of the freeze–thaw gene family members at their target locus could potentially result in an inadvertent disruption to the local gene expression via secondary effects from the spatial positioning of genes, we decided to perform a screen of the non-essential genes within this set to identify these effects.

**Figure 5:**
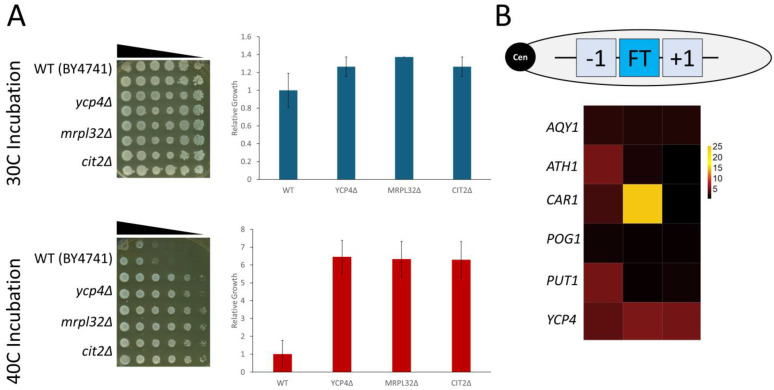
Phenotypic screen for the disruption of genes based on the proximal integration of the *KanMX6* cassette across each freeze–thaw target locus. (**A**) One set of representative pairs of spotting assay plates depicting the growth at the *YCP4* locus at 30 °C versus 40 °C (*left panels*) and the quantified data from all growth assays performed at this locus (*right panels*). (**B**) A schematic of the gene order found at each freeze–thaw locus, FT = freeze–thaw gene, −1 the adjacent ORF on the centromere side, and +1 = the adjacent gene on the non-centromere (e.g., the telomere) side (*top*), and a heatmap depicting the average, relative growth seen at each locus (the data represent the growth at 40 °C relative to the growth at 30 °C). The FT gene at each locus is listed on the left-hand side of the heatmap. The graph of the *YCP4* locus was generated in Excel, and the error bars represent the S.E.M. The heatmap was generated in R.

The most penetrant thermal phenotype associated with the freeze–thaw gene family is the resistance to elevated temperatures compared to that of a wild-type strain. Every viable null mutant was screened for increased growth at 40 °C in strains that harbor the KanMX6 antibiotic resistance cassette in place of the targeted ORF. The growth of each deletion strain, as well as the wild-type strain, was quantified at both ideal (30 °C) and elevated (40 °C) temperatures (Figure 5A, left panels). Multiple replicates were quantified and averaged together, with representative graphs for the *YPC4* locus shown (Figure 5A, right panels). Our data show that the deletion of *CIT2* at this temperature results in a decrease in sensitivity compared to the wild type, consistent with previous reports [54]. Surprisingly, the *MRPL32* gene, which flanks *YCP4* on the opposite side of the chromosome, also demonstrated a reduced sensitivity when incubated at this elevated temperature—which we report here for the first time.

The complete results of this screen revealed that there are several deletion mutants that exhibit a reduced sensitivity to growth at the elevated temperature (Figure 5B). The profiling of the *ATH1*, *CAR1*, *PUT1*, and *YCP4* loci resulted in the identification of null mutants with a reduced sensitivity to growth at 40 °C. Only the *AQY1* and *POG1* loci exhibited minimal change under these specific conditions.

### 2.7. Gene Expression Analysis Throughout the YCP4 Locus by Real-Time PCR

As our phenotypic screen at the *YCP4* locus resulted in the observation that the deletion of either of the genes that flank *YCP4* resulted in an increased resistance to growth at 40 °C, we focused our analysis on this locus specifically. One possibility for this phenotype could be the integration of the *KAN^R^* antibiotic resistance cassette at *MRPL32* or *CIT2* disrupts transcription at the *YCP4* locus, even if that site is itself unchanged by the integration. Gene expression profiling was performed on steady-state cultures of yeast that were captured in their logarithmic growth phase. Expression was obtained for *YCP4*, *KAN^R^*, and *EBP2* (a non-related, negative control gene) and was normalized to the expression of *ACT1* as previously described [27].

**Figure 6:**
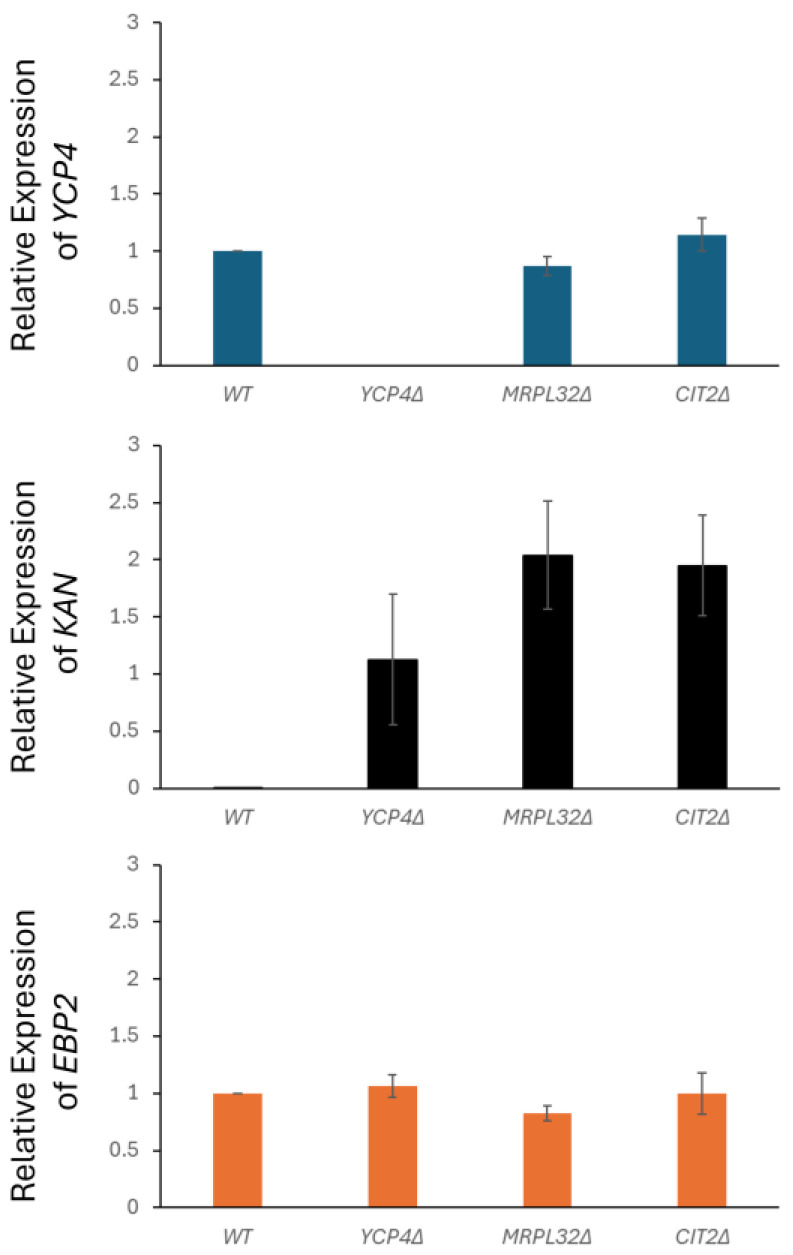
Gene expression analysis and transcriptional profiling following genomic modification across the *YCP4* locus. Real-time PCR was performed to monitor the relative gene expression levels of *YCP4* (*top*), *KAN^R^* (*middle*), and *EBP2* (*bottom*) normalized to the *ACT1* transcript. Bar graphs represent averages of transcripts using the 2^−∆∆Ct^ method, and the error bars are (+/−) S.E.M.

Our results indicate that the level of expression of *YCP4* does not change significantly upon the deletion of the *MRPL32* or *CIT2* gene using the *KAN^R^* selectable marker compared to the levels of expression in the isogenic, wild-type strain (Figure 6). Comparison of the expression of the *KAN^R^* construct in each of the deletion mutants did result in the observation that integration at both the *MRPL32* and *CIT2* had a marked, considerable increase in expression compared to the levels seen at the *YCP4* locus. The transcript levels that we measured in both strains were almost double the levels of expression, although they did not cross the threshold into being statistically significant (e.g., *p* > 0.05). The expression of *EBP2*, which is a gene that is involved in the ribosome biosynthesis and biogenesis gene family, was also measured as a control—and it did not exhibit a similar change in transcription. Overall, this observation is significant, as the constructs that were used to produce each mutant identified were identical (e.g., promoters, CDS, etc.). The fact that the expression varies this much indicates that the consequences of targeted integration at this locus are susceptible to position effects.

### 2.8. Conservation of Synteny and Transcriptional Analysis of the YCP4 Gene in Divergent Yeasts

**Figure 7:**
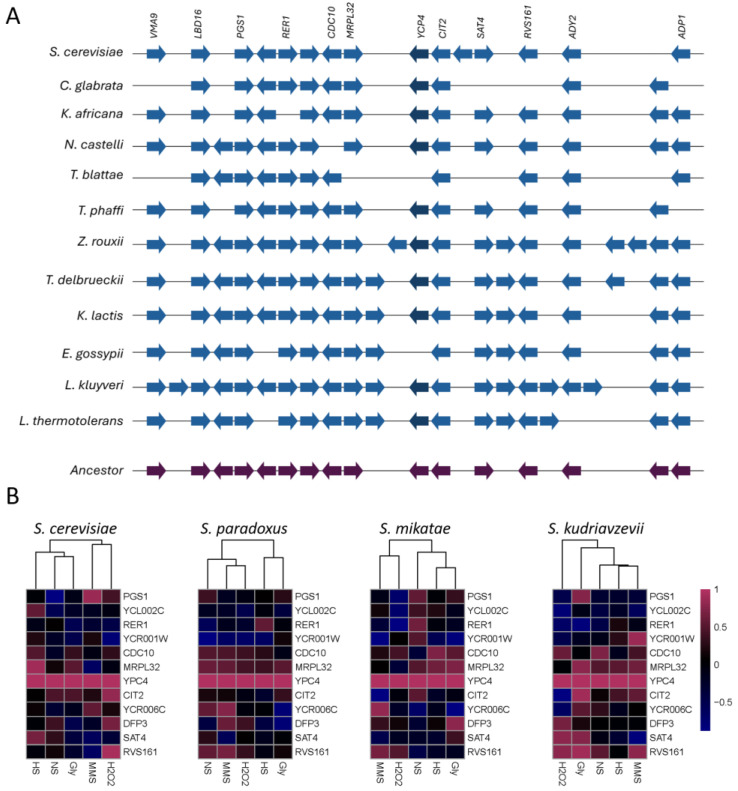
The conservation of synteny and patterns of transcriptional expression across the *YCP4* locus in evolutionarily divergent fungal lineages. (**A**) Syntenic relationships were compared to the genes and open reading frames flanking *YCP4* on both sides. The *YCP4* gene is highlighted by a darker blue color than the rest of the genes throughout, and it is used as the anchoring point for comparison. Conservation genes are aligned vertically, with gaps indicating a lack of conservation and the arrows representing the identified genes. The reconstructed locus of the ancestral relative of these lineages is indicated by the purple coloring at the bottom of the figure. (**B**) Gene expression profiles were extracted, and the PCC was determined for *YCP4* compared to each of the indicated genes that flank it on either side of the chromosome. Heatmaps were generated in R, with clustering to allow comparisons in the response to specific stressors. The gene order has been maintained throughout.

To broaden our understanding of the position effects at this locus, and their potential implications for evolutionarily diverse fungal organisms, we compared the conservation of the genomic structure and syntenic relationships were analyzed (Figure 7A). The *YCP4* ORF is broadly conserved throughout the divergent fungal lineages that were included in our analysis and is predicted to have been present in the genome of a reconstructed ancestral organism. It has been lost in both *T. blattae* and *E. gossypii* but has been identified in each of the other fungi analyzed.

The *CIT2* gene flanks *YCP4* in the direction of the telomere and is completely conserved in all species included in this analysis. *CIT2* is one of the few genes that are present across all the divergent lineages analyzed—the only other genes that have absolute conservation are the more distantly located *RER1*, *PGS1*, and *LBD16*—each of which is found on the opposite chromosomal arm from the *YCP4* locus, separated by the centromere. The *MRPL32* gene flanks *YCP4* in the centromeric direction. This genomic arrangement is conserved in closely related lineages and are predicted to have been present in the reconstructed ancestor; however, it has been interrupted by several insertional events and this spacing is not maintained. Overall, throughout this region, our analysis found that there were few instances of gene insertions across this locus—though the incidences increased as evolutionary divergence increased—compared to gene loss.

We selected three closely related fungi for transcriptional analysis and comparison to *S. cerevisiae*. Gene expression datasets measuring the transcriptional response to stress and nutrient limitations were obtained for *S. paradoxus*, *S. mikatae*, and *S. kudriavzevii*, as well as *S. cerevisiae* from the Gene Expression Omnibus [55]. Transcription profiles were extracted for the genes throughout this locus, and the correlation in transcription (PCC) was determined by comparing *YCP4* to each neighboring gene. These were visualized using a heatmap and the different stressor conditions were allowed to cluster, although the gene order was maintained (Figure 7B). This analysis found that the correlations—and the degree of these correlations—throughout this locus differed in readily identifiable ways.

## 3. Materials and Methods

### 3.1. Identification and Curation of Saccharomyces Freeze–Thaw Tolerance Gene Set

The gene set utilized in this study was identified using the phenotype query ‘freeze thaw resistance: increased’ downloaded from the ‘*Saccharomyces* Genome Database’, which was accessed in January 2024 [37]. The list of identified entries was subsequently paired down to focus on annotations for null gene entries that were viable in a haploid genetic background, and duplicate entries were consolidated. Each locus corresponding to the identified genes was downloaded, used to map the gene set to their chromosomes, and used to create a map of the surrounding genetic region using the maps created and previously described by Cherry et al. [56].

### 3.2. Yeast Strains, Culture Conditions, and Media

All yeast strains utilized in this study were obtained from the haploid Yeast Knock out Collection (Horizon Discovery, Cambridge, UK) and the list of complete genotypes is provided in the Supplementary Materials that accompany this manuscript (Supplementary Table S1). Strains were cultured using enriched media (1% Yeast extract, 2% Peptone, 40 mg/L Adenine, and 2% Dextrose), and they were grown at 30 °C with shaking at 185 r.p.m.

### 3.3. Growth Analysis and Spotting Assays

Cultures of yeast grew overnight until they reached saturation. A 200-microliter volume of each yeast culture, which contained an equal number of cells, was collected by gentle centrifugation (3 min at 1000 r.c.f. at room temperature), washed once with ddH_2_O, and resuspended into a final volume of 200-microliters of ddH_2_O for dilution, freezing, and plating. At that point, the cultures were incubated at the indicated freezing temperatures for each time point analyzed. The freezing process occurred by placing the strains at the indicated temperatures without the addition of a cryoprotectant (there was no snap freezing performed, such as in liquid nitrogen or on dry ice). Thawing occurred at ambient temperature, and the cells were diluted and plated as soon as they completed the thawing process. Growth assays were performed by replica plating, whereby 5-fold serial dilutions of each sample were created, and platings were made using a pin spotting apparatus. The plates were then allowed to dry at an ambient temperature for ~15 min before inversion and incubation at the indicated temperatures. The plates were grown 48–72 h prior to imaging and analysis. The data were compiled and analyzed in Excel, which was used to calculate the graphs and heatmaps were generated in R.

### 3.4. Computational Analysis of Gene Expression

The gene expression profiles for each gene within the freeze–thaw set was extracted from microarray datasets that profiled synchronized cycling cells and cells responding to multiple environmental stressors. The microarray expression profiles for cycling cells can be found at the Gene Expression Omnibus as reference series GSE22. The cells were initially synchronized using alpha-factor, which was then washed away to release the cells from a G_1_/S block in their cell cycle [57]. The stress responses analyzed included heat shock from 25 °C to 37 °C, temperature shift from 29 °C to 33 °C, temperature shift from 30 °C to 37 °C, 0.3 mM hydrogen peroxide treatment, 2.5 mM and 1.0 mM menadione bisulfite, 1.5 mM diamide treatment, 2.5 mM dithiothrietol (DTT), 1.0M sorbitol hyperosmotic shock, and hypoosmotic shock (growth in 1.0M sorbitol and shift to 0.0M sorbitol) [47]. The microarray data can be found at the Gene Expression Omnibus as reference series GSE18. The datasets were analyzed in Excel, which was used to calculate Pearson’s correlation coefficient (PCC) between each gene that was analyzed. Graphs were generated in Excel and heatmaps were generated in R.

### 3.5. RNA Isolation, Purification, and Generation of cDNA

Cultures of yeast grew overnight, and RNA was harvested at an early to mid-log phase of growth (O.D._600_ = 0.25–0.8) using the Fungal/Bacterial RNA extraction kit following the manufacturer’s instructions with the following specifications (Zymo Research, Tustin, CA, USA). A sample of fresh cells that weighed between 50 and 60 mg was used and lysed by using a Vortex Genie bead-beater for 15 min. The manufacturer’s protocol was followed exactly, with the addition of an in-column DNA digestion with a one-hour DNAse I treatment (Zymo Research, CA, USA). The final RNA samples were eluted into a volume of 35 µL DNAse/RNAse-free water and were quantified using a nanodrop spectrophotometer. Degradation was checked by agarose gel electrophoresis, and only samples that retained the presence of intact rRNA bands were used for further analysis.

cDNA for gene expression analysis was generated for each sample utilizing the Zymoscript RT PreMix Kit (Zymo Research, CA, USA). Reverse transcription reactions were set up using between 1 and 2 µg total RNA in a 20 μL reaction, using the recommended reaction conditions and incubation parameters, with no changes at any step.

### 3.6. End-Point PCR, Real-Time PCR, and Transcription Analysis

To test for the efficacy of both the DNAse I treatment (to completely remove any residual gDNA from the RNA samples) and the reverse-transcription reactions (to produce cDNA for gene expression analysis) an end-point PCR was run using PCR primers targeting the *ACT1* coding sequence. At each step of the process the success of the process was tested and verified via end-point PCR. The samples were utilized as the template for a 20 µL PCR reaction using the ZymoTaq Premix (Zymo Research, CA, USA). The cycling parameters for verification followed the manufacturer’s recommendations, with an annealing temperature of 54 °C and an extension time of 30 s for 25 cycles. DNAse treatment was considered a success if the undiluted RNA failed to amplify any bands compared to a positive, gDNA control reaction. The RT reaction was considered a success if the presence of the *ACT1* amplicon was produced by an end-point PCR reaction following generation of the cDNA. Real-time PCR reactions were performed in a final volume of 20 µL, using the iTaq Universal SYBR Green Supermix (Biorad Laboratories, Hercules, CA, USA) for the reactions performed according to the manufacturer’s recommended conditions. The cycling conditions used were a three-step program to allow primer annealing at 55 °C for 15 s prior to extension and quantification. The cycling conditions were run for 45 cycles before a melt curve was obtained, and only reactions that resulted in a single amplicon were used in the final analysis. Each series of reactions was performed in quadruplicate. The final analysis and the determination of gene expression were performed as previously described using the 2^−∆∆Ct^ methodology normalized to actin expression [58]. The complete list of PCR primers used in this study can be found in the Supplementary Materials (Supplementary Table S2).

### 3.7. The Conservation of Synteny and Transcription Across the YCP4 Locus in Divergent Yeast Species

Analysis of the conservation of synteny in related and divergent fungal lineages was obtained from the Yeast Gene Order Browser for all organisms [59]. Expression analyses for *S. cerevisiae*, *S. paradoxus*, *S. mikatae*, and *S. kudriavzevii* were all obtained from the Gene Expression Omnibus (accession number GSE3406), and have been previously described elsewhere [55].

## 4. Conclusions

In this study, we have provided a systematic characterization of the effects of the deletion of the six genes that have been linked to increased survival following a freeze–thaw cycle in budding yeast. Our experimental design utilized the standard genetic background of the BY4741 laboratory strain, and we identified the links between *ATH1*, *POG1*, *CAR1*, *AQY1*, and *YCP4* extended to this genetic background and are linked to increased survival to both decreased and increased temperatures. Furthermore, the use of the KanMX6 antibiotic resistance cassette for gene deletion in this genetic background results in a decrease in the sensitivity of these mutants to elevated temperatures upon integration at multiple sites.

Our follow-up analysis, where we measured the level of transcription across the *YCP4* genomic locus, revealed that the integration of the KanMX6 reporter construct does not disrupt transcription at this locus in a significant way. We should note, however, that this does not rule out the possibility that under other conditions this is the case; however, we only focused our analysis on standard growth conditions (enriched media, 2% glucose) during the period when the cultures were dividing at the maximum rate (log phase cultures). As this is the case, we could rule out one potential model that could explain this finding—this does not appear to be linked to transcriptional disruption that occurs at many loci and is called the ‘Neighboring Gene Effect’ [32,33]. We could not rule out a more recently characterized model—that this is likely the result of secondary effects—such as the titration of the pool of ribosomes from endogenous mRNAs to the KanMX6 transcript decreasing the abundance of specific proteins in a complex manner [60]. There is a finite pool of ribosomes that are available within the cell to mediate protein synthesis, and the integration of the KanMX6 cassette as a reporter has been engineered for high levels of expression [61,62]. Replacing the endogenous gene with this highly expressed reporter introduces the corresponding mRNA at a level that is almost always expressed at a significantly higher level than the gene that was replaced—the KanMX6 mRNA has been quantified, and it is in the 98th percentile in its abundance [60]. As this is the case, there are several non-mutually exclusive explanations that we can envision for our observations: that the pool of ribosomes is titrated in a way that limits the translation of a specific protein that leads to an indirect change in phenotype, that the levels of mRNA differ under specific non-standard growth conditions, and that the mRNA levels are affected in a significant manner that does not strictly adhere to the strict *p*-value cutoff chosen for significance. It is the author’s belief that it is most likely a combination of all three potential mechanisms.

Furthermore, our work has implications that may extend far beyond this particular strain and this particular organism—as the conservation of synteny and expressional correlations in evolutionarily divergent lineages can provide a resource that can be applied to other, non-traditional yeasts that are increasingly being engineered in this way, including *Rhodotorula glutinis*, *Lipomyces starkeyi*, and *Yarrowia lipolytica*, to name a promising select few organisms [63,64,65].

## Figures and Tables

**Table 1:** Annotated Genes Resulting in Increased Freeze–Thaw Tolerance Following Deletion.

Gene	Identifier	Background	Functional Annotation and References
*AQY1*	YPR192W	SK1	Spore-specific water channel [40]
*ATH1*	YPR026W	SEY6210	Acid trehalase [41]
*CAR1*	YPL111W	Other	Arginase [42]
*POG1*	YIL122W	Other	DNA-binding TF activator [43]
*PUT1*	YLR142W	S288C; Other	Proline oxidase (mitochondrial) [38,44,45]
*YCP4*	YCR004C	S288C	Protein of unknown function (mitochondria) [39]

**Table 2:** Genes that flank the freeze–thaw family.

Freeze–Thaw Gene	+1 Neighbor ^1^	−1 Neighbor ^2^
*AQY1*	*HPA1*	*QCR2*
*ATH1*	*YPR027C*	*CCL1* *
*CAR1*	*PEX25*	*GDE1*
*POG1*	*SIM1*	*QDR2*
*PUT1*	*DPH6*	*RRN5* *
*YCP4*	*CIT2*	*MRPL32*

^1^ Telomeric direction; ^2^ Centromeric direction; * Essential genes.

## Data Availability

All data utilized within this study are described in the materials and methods, where the details of accession numbers to specific datasets can be found.

## Supplementary Materials

The supporting information can be downloaded at: https://www.mdpi.com/article/10.3390/ijms26052149/s1.
