# Supplementary material for: Systematic Characterization and Analysis of the Freeze–Thaw Tolerance Gene Set in the Budding Yeast, Saccharomyces cerevisiae
Source: Int J Mol Sci. 2025 Feb 27;26(5):2149. doi: 10.3390/ijms26052149 (PMC11900043; doi:10.3390/ijms26052149)

Hartnett et al. 2025 Supplemental files:

Supplementary Table S1: Complete genotypes of yeast strains used in this study

Supplementary Table S2: PCR primers used in this study

Supplementary Figure S1: Strain sensitivity to -80C freeze-thaw stress followed by incubation at 30C

Supplementary Figure S2: Strain sensitivity to -80C freeze-thaw stress and by incubation at 40C

Supplementary Table S1: Complete genotypes of yeast strains used in this study

Strain name	Complete genotype
Wild-type (BY4741)	*MATa his3Δ1 leu2Δ0 met15Δ0 ura3Δ0*
pog1Δ	*MATa his3Δ1 leu2Δ0 met15Δ0 ura3Δ0 pog1::KANR*
ath1Δ	*MATa his3Δ1 leu2Δ0 met15Δ0 ura3Δ0 ath1::KANR*
put1Δ	*MATa his3Δ1 leu2Δ0 met15Δ0 ura3Δ0 put1::KANR*
ycp4Δ	*MATa his3Δ1 leu2Δ0 met15Δ0 ura3Δ0 ycp4::KANR*
car1Δ	*MATa his3Δ1 leu2Δ0 met15Δ0 ura3Δ0 car1::KANR*
aqy1Δ	*MATa his3Δ1 leu2Δ0 met15Δ0 ura3Δ0 aqy1::KANR*
hpa2Δ	*MATa his3Δ1 leu2Δ0 met15Δ0 ura3Δ0 hpa2::KANR*
qcr2Δ	*MATa his3Δ1 leu2Δ0 met15Δ0 ura3Δ0 qcr2::KANR*
ypr0027CΔ	*MATa his3Δ1 leu2Δ0 met15Δ0 ura3Δ0 ypr0027C::KANR*
gde2Δ	*MATa his3Δ1 leu2Δ0 met15Δ0 ura3Δ0 gde1::KANR*
pex25Δ	*MATa his3Δ1 leu2Δ0 met15Δ0 ura3Δ0 pex25::KANR*
qdr2Δ	*MATa his3Δ1 leu2Δ0 met15Δ0 ura3Δ0 qdr2::KANR*
sim1Δ	*MATa his3Δ1 leu2Δ0 met15Δ0 ura3Δ0 sim1::KANR*
dph6Δ	*MATa his3Δ1 leu2Δ0 met15Δ0 ura3Δ0 dph6::KANR*
mrpl32Δ	*MATa his3Δ1 leu2Δ0 met15Δ0 ura3Δ0 mrpl32::KANR*
cit2Δ	*MATa his3Δ1 leu2Δ0 met15Δ0 ura3Δ0 cit2::KANR*

Supplementary Table S2: PCR primers used in this study

PCR application	Primer name	Sequence (5′ to 3′)
End point	*Act1* – CDS FP	GTTTCCATCCAAGCCGTTTTGTC
End point	*Act1* – CDS RP	CGACATCACACTTCATGATGGAG
Real time	*YCP4* – CDS FP	GACCTGACGGCTCAAGAACT
Real time	*YCP4* – CDS RP	GCCGCATCAGAAGTAGTGGT
Real time	*KanMX6* – CDS FP	TCACCGGATTCAGTCGTCAC
Real time	*KanMX6* – CDS RP	GGAGAAAACTCACCGAGGCA
Real time	*ACT1* – CDS FP	ATCGTTATGTCCGGTGGTACC
Real time	*ACT1* – CDS RP	TGGAAGATGGAGCCAAAGC
Real time	*EBP2* – CDS FP	AACGCTACCTTACAGAAACG
Real time	*EBP2* – CDS RP	TCCGTTAGGCCTGCCTCTATCGAA

Supplementary Figure S1: Growth analysis of the freeze-thaw phenotype seen at -80C in the BY4701 genetic background. Cultures of yeast were grown to saturation, washed with ddH_2O, and frozen for the indicated period of time before being thawed, serially-diluted 5-fold, and plated on enriched media. A) Representative plates depicting the growth of each strain performed in duplicate (each strain is plated as two rows representing technical replicates). B) Quantification of the growth phenotypes seen for multiple biological (n=3) and technical (n=6) replicates were averaged and plotted in the graphs. Error bars represent the S.E.M. There were no significant differences determined by a student T-test.

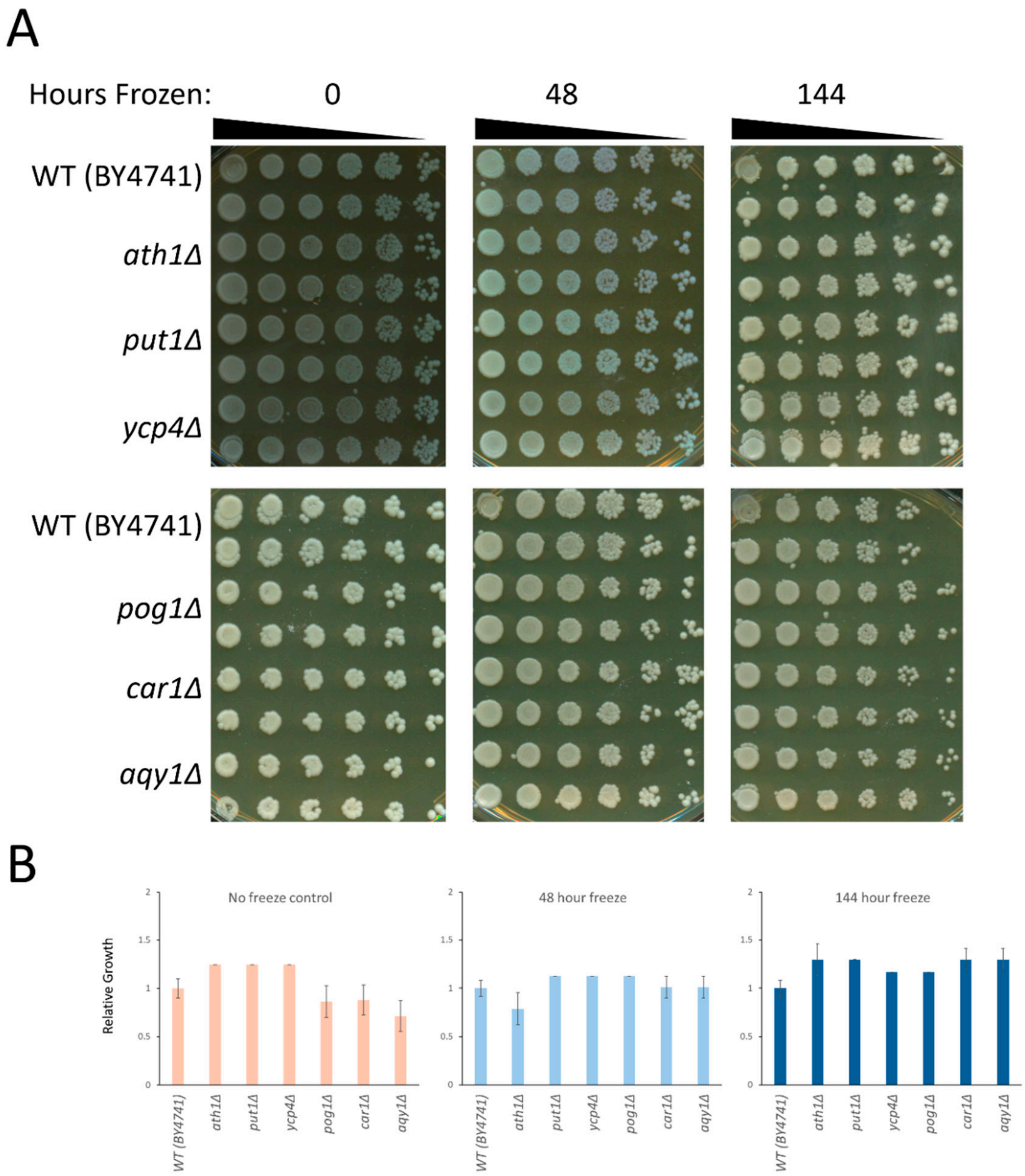

Supplementary Figure S2: Comprehensive growth analysis of the thermal sensitivity phenotypes seen in the BY4701 genetic background. Strains were incubated and growth was assayed at either optimal (control, 30C) or elevated(40C) temperatures with and without freezing and incubation at -20C. A) Representative plates depicting the growth of each strain performed in duplicate (each strain is plated as two rows representing technical replicates). B) Quantification of the growth phenotypes seen for multiple biological (n=3) and technical (n=6) replicates were averaged and plotted in the graphs. Error bars represent the S.E.M. Significant differences were determined by a student T-test, comparing the growth of each mutant to growth of the WT strain. Significant differences in the growth observed are indicated on the graph (*** = p<0.05).

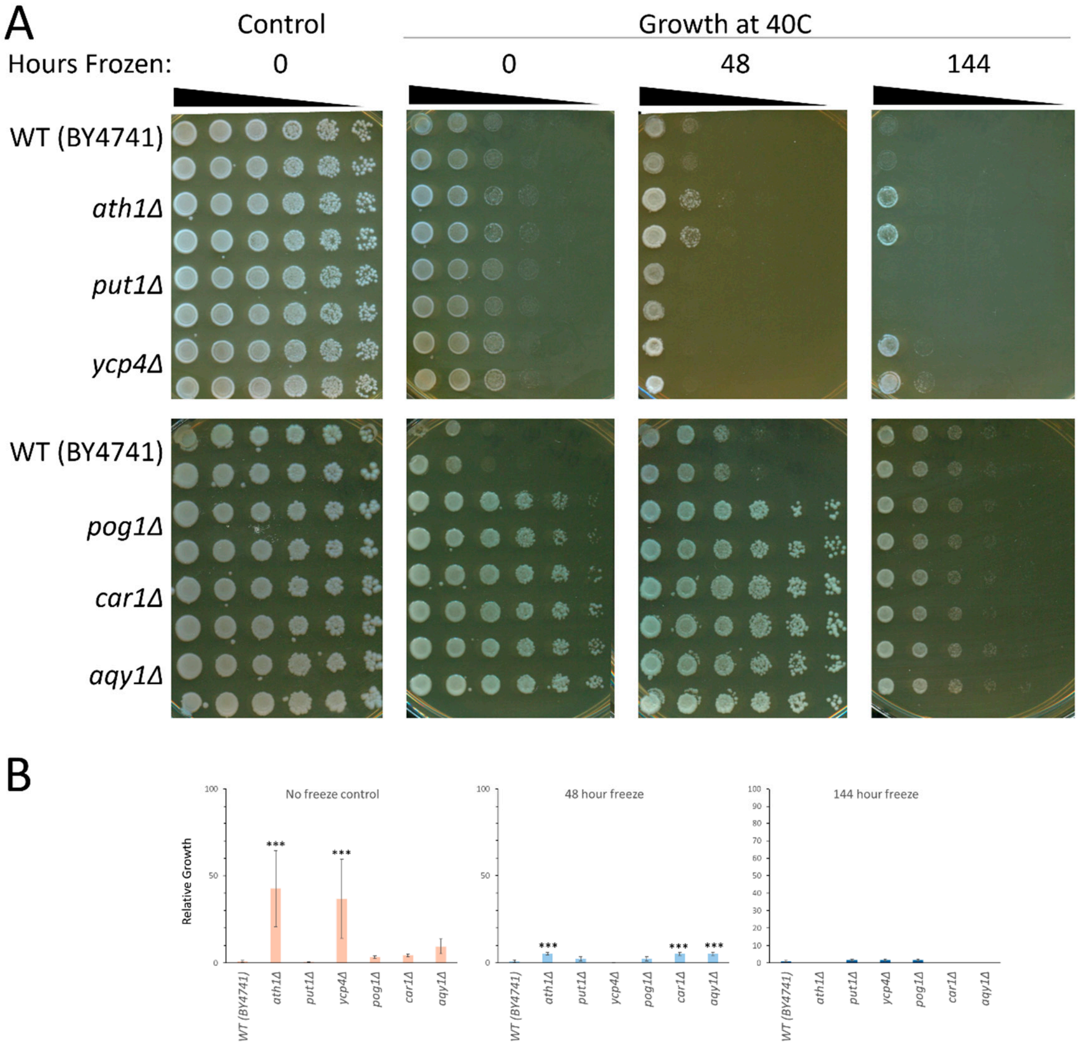

Supplement: Supplementary file 1 [file ijms-26-02149-s001.zip › ijms-3488064-supplementary.pdf]
